# The interplay of inflammation and placenta in maternal diabetes: insights into Hofbauer cell expression patterns

**DOI:** 10.3389/fimmu.2024.1386528

**Published:** 2024-03-25

**Authors:** Zdenek Tauber, Adela Burianova, Katerina Koubova, Max Mrstik, Marie Jirkovska, Katerina Cizkova

**Affiliations:** ^1^ Department of Histology and Embryology, Faculty of Medicine and Dentistry, Palacky University, Olomouc, Czechia; ^2^ Institute of Histology and Embryology, First Faculty of Medicine, Charles University in Prague, Prague, Czechia

**Keywords:** hofbauer cells, placental inflammation, type 1 diabetes mellitus, gestational diabetes mellitus, CYP epoxygenases, soluble epoxide hydrolase

## Abstract

**Introduction:**

Inflammation of the placenta is harmful to both the fetus and the mother. Inflammation is strongly associated with diabetes, a common complication of pregnancy. Hofbauer cells (HBCs), unique immune system cells of fetal origin in the placenta, play complex roles, including growth of placental villi and their branching, stromal remodelling, and angiogenesis.

**Methods:**

Our study investigated the expression of IL-1β, IL-10, CYP2C8, CYP2C9, CYP2J2 and sEH in HBCs from patients with type 1 diabetes mellitus (T1DM) and gestational diabetes mellitus (GDM) compared to healthy controls using immunohistochemistry. We also assessed the structure of the villus stroma using Masson´s trichrome.

**Results:**

In T1DM, HBCs showed inflammatory activation characterised by increased IL-1β and decreased CYP epoxygenase expression compared to normal placentas. Conversely, significant inflammation in HBCs appeared less likely in GDM, as levels of IL-1β and CYP epoxygenases remained stable compared to normal placentas. However, GDM showed a significant increase in sEH expression. Both types of diabetes showed delayed placental villous maturation and hypovascularisation, with GDM showing a more pronounced effect.

**Conclusion:**

The expression profiles of IL-1β, CYP epoxygenases and sEH significantlly differ between controls and diabetic placentas and between T1DM and GDM. These facts suggest an association of the CYP epoxygenase-EETs-sEH axis with IL-1β expression as well as villous stromal hypovascularisation. Given the stable high expression of IL-10 in both controls and both types of diabetes, it appears that immune tolerance is maintained in HBCs.

## Introduction

The placenta is an important organ that provides the link between the mother and the developing fetus during pregnancy. It is not only important for the nutrition of the fetus, but also for the establishment of immunological tolerance, a prerequisite for a successful pregnancy. The unique composition and interplay of humoral and cellular factors at the feto-maternal interface contribute significantly to this. Macrophages are the second most abundant immune cells in the human placenta. The current view is that they can be classified into the following groups according to their origin, location and functional characteristics: placenta-associated maternal macrophages (PAMMs) and Hofbauer cells (1).

Hofbauer cells (HBCs) are the only known elements of the placental immune system that are of fetal origin. They are most commonly found in the stroma of placental villi, usually close to blood vessels or the superficial trophoblast (2). They are thought to originate from mesenchymal progenitor cells of the chorionic villi and yolk sac at early stages of embryonic development and from fetal haematopoietic monocytes at later stages of development (2–9). HBCs have been identified in the placenta as early as day 18 of intrauterine development and persist until the time of delivery, but their number has been shown to gradually decrease from 4-5 months of development (7). They are relatively large (10-30 µm) cells with an abundant, highly vacuolated and granular cytoplasm (10). Initially described as oval cell elements, HBCs of star and spindle shape have subsequently been identified (7, 10–12).

HBCs are considered to be alternatively activated M2 macrophages (13–17). This corresponds to the expression of CD163, CD209, CD206 antigens and the production of IL-10, TGFβ, VEGF-A, FGF-2, osteopontin, MMP-9 and TIMP-1 (1, 18) HBCs fulfil a variety of functions during pregnancy. They participate in villous growth and branching, in remodelling of extracellular matrix and they also promote angiogenesis and vasculogenesis. They are involved in the maintenance of immunological tolerance at the feto-maternal interface, in elimination of apoptotic cells and they also exhibit antimicrobial activity (1, 2, 19). Interestingly, HBCs, similar to classically activated M1 macrophages, are able to produce IL-1β, IL-8, CCl-2,3 and 4, factors typically associated with inflammation (1, 20).

Hyperglycaemia in pregnancy is a common complication with an incidence of 16.7% of live births according to the International Diabetes Federation. It can manifest itself as diabetes mellitus in pregnancy, also known as type 1 (T1DM) or type 2 (T2DM) pregestational diabetes. Most cases of hyperglycaemia in pregnancy (84%) are gestational diabetes mellitus (GDM) (21). In normal pregnancy, a physiological state of insulin resistance directs maternal nutrients preferentially to the feto-placental unit, allowing for adequate fetal growth. In GDM, insulin resistance is more severe and disrupts the intrauterine environment, resulting in accelerated fetal development with an increased risk of macrosomia (22, 23). In contrast, T1DM is a deficiency in insulin production by the islets of Langerhans as a consequence of their autoimmune damage (24).

Metabolic disorders such as diabetes and obesity are closely associated with systemic inflammation, including placental one (25, 26). The factors that lead to the initiation of the inflammatory response in patients with diabetes are not yet fully understood. However, it is clear from *in vitro* studies that hyperglycaemia stimulates TLR-dependent inflammatory pathways, including TLR2, TLR4 and their downstream molecules, leading to overproduction of proinflammatory cytokines by macrophages (27, 28). This condition contributes to the development of insulin resistance and impaired glucose tolerance, as shown by studies conducted mainly on adipose tissue macrophages and adipocytes (29, 30). In addition to the above, it is also known that hyperglycaemia in pregnancy is associated with delayed maturation of placental villi (31, 32).

The phenotype of macrophages and their cytokine production is also related to the level of epoxyeicosatrienoic acids (EETs) (29, 33, 34). EETs play a variety of biological roles in the body and are known for their anti-inflammatory features. EETs are derived from arachidonic acid by CYP epoxygenases. Arachidonic acid (AA) is a ω-6 unsaturated fatty acid which is a part of normal diet. Its concentration in fetal plasma is higher than in maternal plasma and it is preferentially transported across the placenta over non-essential fatty acids and its precursor linoleic acid (35, 36). Arachidonic acid is also a component of the phospholipids of cytoplasmic membranes from which it is released by the activity of phospholipase A2 (37). EETs are known to be synthesised in humans mainly by the CYP2C and CYP2J subfamilies. The CYP2C subfamily consists of four enzymes, namely CYP2C8, CYP2C9, CYP2C18 and CYP2C19. CYP2J2 is the only member of the CYP2J subfamily in humans. The activity of CYP2C and CYP2J2 results in a total of four regioisomers of EETs, namely 5,6-EET, 8,9-EET, 11,12-EET and 14,15-EET, the latter two being the most abundant. Individual CYP epoxygenases produce EET regioisomers in different ratios (38, 39). EETs are highly active substances with short biological half-lives. They are either incorporated back into biomembranes or hydrolysed by soluble epoxide hydrolase (sEH) to biologically less active dihydroxyeicosatrienoic acids (DHETs) (40). In addition to CYP epoxygenases, AA can also be metabolised in cells by three different enzymatic pathways, namely cyclooxygenase, lipoxygenase and CYP ω-hydroxylases.

The effect of hyperglycaemia on the biological behaviour of HBCs has been studied to a limited extent. The results are highly contradictory and the possible further consequences for the placenta as a whole remain unknown (28, 41). Because EETs seem to play a role in the pathophysiology of diabetes, the aim of this study was to describe the expression profiles of EET-generating (CYP2C8, CYP2C9, CYP2J2) and EET-degrading (sEH) enzymes in HBCs from patients with T1DM and GDM compared to healthy controls. Moreover, it is known that EETs are involved in immunomodulation and inflammation, thus we also detected IL-10 and IL-1β. In addition, HBCs are known to play a role in extracellular matrix remodelling and angiogenesis, and simultaneously, EETs are pro-angiogenic molecules. It is also known that diabetes is associated with delayed maturation of placental villi and changes in vasculature. We therefore investigated whether the IHC profiles obtained correspond to structural changes in the placental villous stroma.

## Material and methods

### Tissue samples

A total of 54 formalin-fixed, paraffin-embedded samples of term placentas from healthy controls (n = 18) and patients with type 1 diabetes mellitus (T1DM, n = 22) and gestational diabetes mellitus (GDM, n = 14) were used. The samples were collected in 1999-2011 at the Department of Gynaecology, Obstetrics and Neonatology, First Faculty of Medicine and General University Hospital in Prague. It is a retrospective study using archival material. Routine hematoxylin-eosin staining was performed on all samples used to assess tissue integrity. All available characteristics of the samples are summarised in **Table 1**. The use of the samples was approved by the Ethics Committee of the University Hospital and the Faculty of Medicine and Dentristy, Palacký University, Olomouc (ref. no. 151/23).

**Table 1:** Characteristics of patient samples.

type 1 diabetes mellitus (T1DM)								
No.	mother´s age (years)	duration of diabetes (years)		GlyHb%	gestation week	delivery	placenta (g)	newborn (gender/g/cm)
1	34	20		7.7	38	C-section	655	F/3630/49
2	35	6		3.8	38	C-section	645	M/3680/52
3	33	15		3.7	40	C-section	620	M/3790/51
4	21	7		5.8	37	spontaneous	550	M/2920/47
5	36	17		4.0	39	C-section	770	F/4070/53
6	23	1		3.1	39	spontaneous	490	F/3170/49
7	36	15		3.3	38	C-section	475	F/2860/46
8	31	13		4.5	37	C-section	560	M/3570/51
9	28	14		4.8	39	C-section	500	M/3390/48
10	24	11		4.7	37	C-section	400	M/2400/42
11	38	27		5.0	38	C-section	680	F/4290/52
12	42	14		4.4	36	C-section	695	M/4200/55
13	31	7		3.8	40	C-section	615	F/2880/47
14	33	10		4.8	39	spontaneous	610	M/3430/49
15	38	4		5.0	35	C-section	310	F/1845/41
16	30	10		4.3	40	spontaneous	560	F/3900/51
17	29	6		7.2	38	C-section	620	F/4480/53
18	36	23		4.1	34	C-section	370	F/2230/46
19	34	15		3.4	39	spontaneous	525	M/3210/49
20	34	5		9.4	32	C-section	754	M/2900/45
21	30	21		5.2	32	C-section	845	M/3260/50
22	22	20		7.7	38	C-section	760	M/3950/49
gestational diabetes (GDM)								
No.	mother´s age (years)	treatment			gestation week	delivery	placenta (g)	newborn (gender/g/cm)
23	27	diet			39	C-section	450	M/3010/50
24	28	diet			38	spontaneous	570	M/3140/43
25	28	–			39	C-section	1000	M/3760/51
26	27	insulin			40	spontaneous	520	M/3250/50
27	29	insulin			39	spontaneous	560	M/3080/50
28	32	diet			41	spontaneous	585	F/3940/54
29	31	diet			39	spontaneous	725	M/3555/53
30	30	insulin			37	spontaneous	500	M/3020/50
31	31	diet			40	spontaneous	550	F/3840/50
32	34	insulin			40	spontaneous	590	F/3280/50
33	37	–			39	spontaneous	570	F/3470/51
34	29	–			38	spontaneous	405	F/2910/47
35	30	insulin			37	spontaneous	476	F/2570/46
36	33	diet			39	spontaneous	340	F/3330/51
controls								
No.	mother´s age (years)		gestation week			delivery	placenta (g)	newborn (gender/g/cm)
37	30		39			C-section	900	M/3700/
38	27		40			C-section	555	F/3030/
39	30		39			C-section	635	M/3870/52
40	24		40			C-section	620	M/3730/53
41	26		38			C-section	435	F/2780/45
42	33		38			C-section	700	M/3470/50
43	37		39			C-section	740	M/4110/53
44	24		38			spontaneous	390	F/2890/50
45	36		38			C-section	770	F/4200/51
46	30		39			spontaneous	600	F/3380/49
47	38		39			spontaneous	535	M/3490/50
48	31		39			C-section	605	M/3560/50
49	23		39			spontaneous	580	F/3520/52
50	31		38			C-section	590	F/3175/50
51	31		39			C-section	603	F/3880/50
52	30		41			C-section	575	M/3180/49
53	30		41			C-section	640	M/3820/52
54	30		39			C-section	460	F/3250/49

### Confirmation of Hofbauer cells in tissue samples

HBCs are the only elements of immune system found within the villous stroma. The presence of HBCs was confirmed in routine staining by their typical morphology and localization. Importantly, there is no marker specific for HBC. HBCs are considered as M2 macrophages. Thus, M2 markers, such as CD206, CD163, or CD209 are suitable for their detection (1, 18). Based on these, we used CD206 for their detection in our study.

### Immunohistochemistry

All proteins of interest were detected in 4 µm thick paraffin sections by two-step indirect immunohistochemistry. Briefly, slides were deparaffinised, hydrated and antigens were unmasked by heat antigen retrieval in citrate buffer pH 6. For CD86 immunostaining, Tris/EDTA buffer pH 9 was used. Non-specific background staining was blocked with Protein Block (Dako) for 30 minutes at room temperature (RT). The samples were then incubated with primary antibodies for 1 hour at RT. The following primary antibodies were used: rabbit polyclonal antibodies against CD206 (Abcam; ab64693) at a dilution of 1:1000, IL-10 (Abcam; ab34843) at a dilution of 1:400, IL-1β (Novus Biologicals, NBP1-19775) at a dilution of 1:100, CYP2C8 (ProteinTech, 16546-1-AP) at a dilution of 1: 50 and CYP2J2 (Abcam, ab151996) at 1:100 dilution and mouse monoclonal antibody against CD86 (Abcam; ab220188) at 1:50 dilution, CYP2C9 (Novus Biologicals, NBP2-01381) at 1:400 dilution and sEH (Santa Cruz, sc166916) at 1:200 dilution. Appropriate dilutions of primary antibodies were determined by staining positive control samples as recommended by the manufacturer. Antibodies were diluted in Dako REALTM Antibody Diluent (Dako). Visualisation was performed using the Mouse/Rabbit PolyDetector DAB HRP Brown Detection System (Bio SB). Tissue sections were counterstained with haematoxylin. Tris buffer (pH 7.6) was used for washing between steps. The samples were then dehydrated and coverslipped. Positive and negative controls were included in the immunostaining of samples to verify the staining process. As a negative control, the primary antibody was replaced with Tris buffer followed by incubation with the detection system.

The semi-quantitative evaluation of the staining intensity under the microscope according to the signal intensity was graded as follows: 0 for negative tissue, 1 for weak signal, 2 for moderate signal and 3 for strong signal. The intensity was evaluated in 100 HBCs in each sample and median of these values represents the final staining intensity of the sample. The samples were evaluated twice at separate times by an experienced histologist. The samples were blinded before evaluation. The results were summarized in graphs. The distribution of staining intensities is shown in the graphs as points (one point represents one sample) and the columns show the medians.

### Masson´s trichrome staining

The collagen and vascular fractions in the placental villi were evaluated after Masson’s trichrome staining. For image analysis, RGB images of 10 different fields of vision with a resolution of 1920x1200 pixels, saved as.tiff, were obtained using an Olympus BX40 light microscope equipped with an Olympus DP71 camera at 200x magnification for each sample. The tissue was carefully evaluated by experienced histologist using ImageJ software. The villous area, collagen area and vascular area of 15 terminal villi from each patient were detected. The binary mask for these structures were saved for measurement. AutoThreshold combined with Analyze Particles function were used to detect villi area. Vascular area was annoted manually. The collagen fraction was detected using Trainable Weka Segmentation plugin, which combines image segmentation and machine learning algorithm (42). The areas of villus, collagen and blood vessels were measured (in pixel unit). The calculation was as follows:


$$collagen\;fraction=\frac{collagen\;area}{\left(villi\;area-vascular\;area\right)}$$

$$vascular\;fraction=\frac{vascular\;area}{villi\;area}$$

The results are summarised in graphs showing the distribution of values in each field of vision (individual points) and the mean ± SD (red and black lines).

### Statistical evaluation

The obtained immunostaining intensities, as well as for trichrome staining, were evaluated by Kruskal-Wallis test to find differences between the studied groups, followed by Dunn’s multiple comparison test at the level of significance p< 0.05 for all test performed. Statistically significant results are marked with an asterisk (*) in the graphs and figures. We used: * for p< 0.05, ** for p< 0.01, *** for p< 0.001 and **** for p< 0.0001. All calculations were performed using GraphPad Prism 8 software.

The contribution of samples characteristics such as mother age, foetal sex, gestation age, delivery, and diagnosis to obtained IHC results were evaluated using ordinal logistic regression. These tests were perfomed by TIBCO Statistica software at the level of significance p< 0.05.

## Results

### Confirmation of HBCs and immunostaining profiles of IL-1β and IL-10

**Figure 1:**
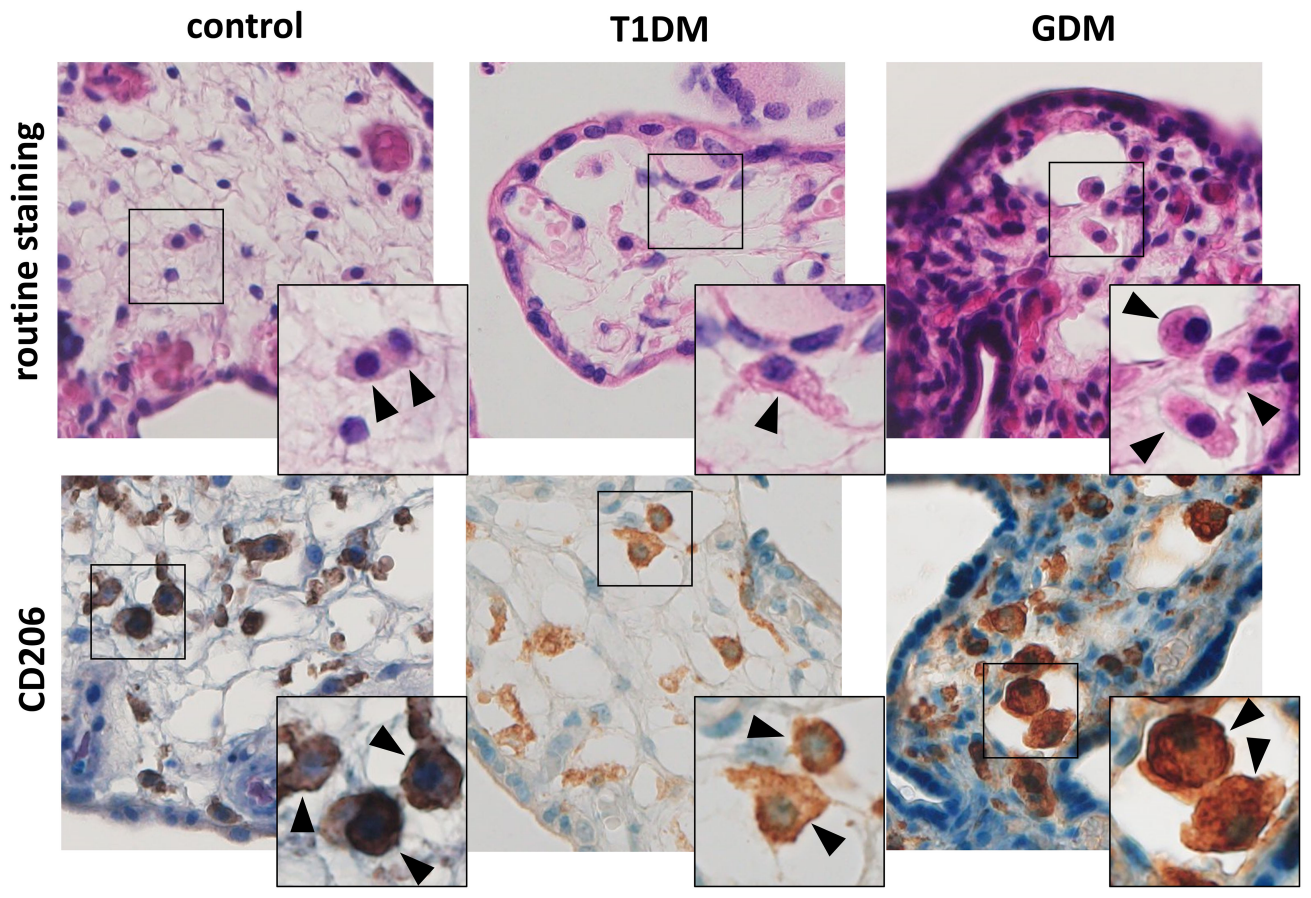
Hofbauer cells in the placenta. The routine staining shows typical morphology and localization of HBCs. Their presence was confirmed using CD206. All microphotographs are magnified 200x, details are 400x. HBCs are marked by arrowheads.

We detected CD206+ cell in villous stroma in all samples tested. These cells showed typical morphology and localization of HBCs (see **Figure 1**). Moreover, all the samples were also negative for CD86, a marker for M1 macrophages (data not shown).

**Figure 2:**
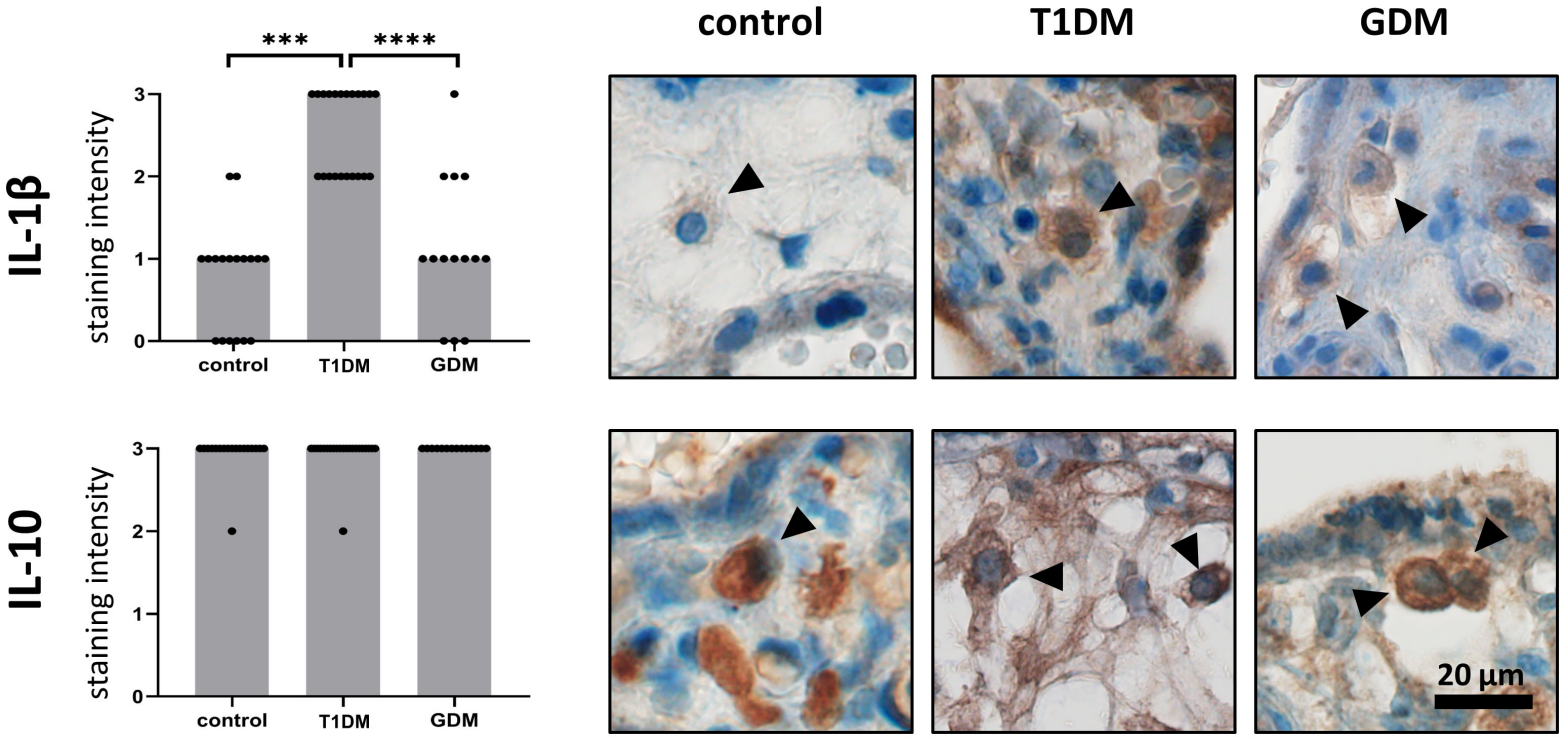
Expression of IL-1b and IL-10 in Hofbauer cells. The total immunohistochemical (IHC) staining profile is shown in the graphs. Micrographs show the representative images of IL-1β and IL-10 in normal term placenta (controls), placenta with insulin-dependent diabetes mellitus (T1DM) and placenta with gestational diabetes mellitus (GDM). Staining intensity was scored as: negative (0), weak (1), moderate (2) and strong (3). The distribution of staining intensities is shown as points on the graphs (one point represents one sample). Columns show medians. Statistically significant results are marked directly in the figure with p-values. Number of samples: controls (n = 18), T1DM (n = 22), GDM (n = 14). Results were evaluated by Kruskal- Wallis followed by Dunn’s multiple comparison test at a significance level of p< 0.05. Significant results are marked with an asterisk (*) directly in the graphs: *** for p< 0.001 and **** for p< 0.0001. All photomicrographs magnified 400x, black line represents 20 μm. Hofbauer cells are marked by arrow heads.

We confirmed the expression of IL-1β and IL-10 in HBCs in normal term, T1DM and GDM placentas. Both interleukins showed cytoplasmic staining, consistent with their expected intracellular localization. The expression levels of the interleukins studied in normal, T1DM and GDM placentas are summarised in **Figure 2** together with representative micrographs. IL-1β showed significant differences between the groups evaluated. Its expression is significantly higher in placenta with T1DM compared to control (p = 0.002) as well as in placenta with GDM (p< 0.0001). There was no difference between control and GDM placenta (p = 0.9688). Surprisingly, the staining intensity of IL-10 showed a very uniform and high expression in all three groups (p = 0.6903, Kruskal-Wallis test).

### Immunostaining profiles of CYP epoxygenases and sEH

**Figure 3:**
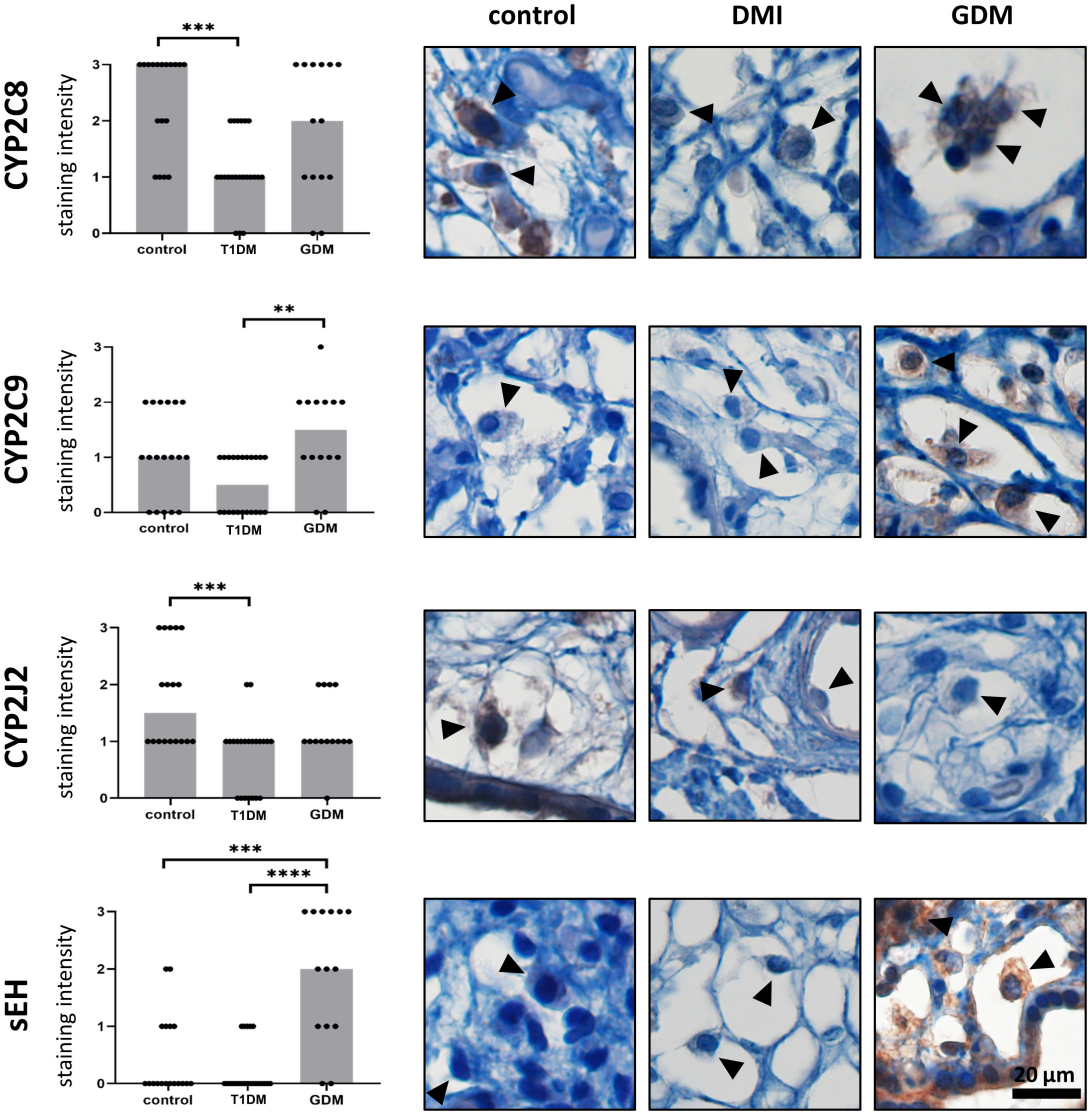
Expression of CYP2C8, CYP2C9, CYP2J2 and sEH in Hofbauer cells. The total immunohistochemical (IHC) staining profile is shown in the graphs. Micrographs show the representative images of the protein of interest in normal term placenta (controls), placenta with insulin dependent diabetes mellitus (T1DM) and placenta with gestational diabetes mellitus (GDM). Staining intensity was scored as: negative (0), weak (1), moderate (2) and strong (3). The distribution of staining intensities is shown as points on the graphs (one point represents one sample). Columns show medians. Statistically significant results are marked directly in the figure with p-values. Number of samples: controls (n = 18), T1DM (n = 22), GDM (n = 14). Results were evaluated by Kruskal-Wallis followed by Dunn’s multiple comparison test at a significance level of p< 0.05. Significant results are marked with an asterisk (*) directly in the graphs: ** for p< 0.01, *** for p< 0.001 and **** for p< 0.0001. All photomicrographs magnified 400x, black line represents 20 μm. Hofbauer cells are marked by arrow heads.

Expression of CYP2C8, CYP2C9, CYP2J2 and sEH was confirmed in HBCs in normal term placenta, T1DM and GDM placenta. All studied antigens showed cytoplasmic staining in accordance with their expected intracellular localisation. The expression levels of the tested CYP epoxygenases and sEH in normal, T1DM and GDM placenta are summarised in **Figure 3** together with representative micrographs.

The highest staining intensity of CYP2C8 was found in controls (median 3) and the lowest in T1DM (median 1). These two groups were statistically different (p = 0.0003). The expression of CYP2C8 in GDM was in the middle of the previous groups. However, there was no significant difference compared to controls (p = 0.4415) or T1DM (p = 0.1086). In addition, CYP2C8 showed the highest staining intensity among the epoxygenases tested in normal term placentas. CYP2C9 also showed the lowest level in T1DM, but the highest median staining intensity was detected in GDM. CYP2C9 staining intensities differed significantly only between T1DM and GDM (p = 0.0030). The difference between controls and T1DM did not reached significance (p = 0.0790). There was no difference between controls and GDM (p = 0.7234). The median CYP2J2 staining intensity was highest in controls (median 1.5) and showed comparable levels in both types of diabetes. CYP2J2 expression was significantly different only between controls and T1DM (p = 0.0006). Comparisons between controls and GDM (p = 0.3502) and between T1DM and GDM (p = 0.2020) were not significant. Expression of sEH was barely detectable in controls and T1DM, but clearly distinguishable in GDM (median 2 vs 0 in controls and T1DM). The expression of sEH differed significantly between GDM and controls (p = 0.0009) and between both types of diabetes (p< 0.0001).

### Influence of sample characteristics on immunohistochemical profiles of studied antigens

In general, the ordinal logistic regression reveals that mother age, gestational age, foetal sex, delivery does not significantly contribute to IHC results. Only the diagnosis significantly contributed to obtained results (see **Table 2**), with only one exeption, namely mother age and CYP2J2 expression.

**Table 2:** Factors affecting immunohistochemical profiles.

		Wald Statistics	p-value
**IL-1beta**	**diagnosis**	**20.6322**	**< 0.0001**
	mother´s age	1.8870	0.1695
	gestation week	0.0435	0.8348
	delivery	1.2590	0.2618
	foetal sex	0.6440	0.4223
**CYP2C8**	**diagnosis**	**9.9729**	**0.0068**
	mother´s age	3.6581	0.0558
	gestation week	0.7867	0.3751
	delivery	0.3010	0.5833
	foetal sex	1.1284	0.2881
**CYP2C9**	**diagnosis**	**8.5217**	**0.0141**
	mother´s age	3.1056	0.0780
	gestation week	0.0036	0.9523
	delivery	0.2078	0.6485
	foetal sex	0.4085	0.5228
**CYP2J2**	**diagnosis**	**14.3997**	**0.0007**
	**mother´s age**	**8.2808**	**0.0040**
	gestation week	3.5801	0.0585
	delivery	0.5688	0.4507
	foetal sex	0.7210	0.3958
**sEH**	**diagnosis**	**17.0155**	**0.0002**
	mother´s age	1.8727	0.1712
	gestation week	1.1486	0.2838
	delivery	1.8359	0.1754
	foetal sex	0.0653	0.7984

Results of ordinal logistic regresion, level of significance: p< 0.05. Significant factors are bold.

### Characterization of villous stroma

**Figure 4:**
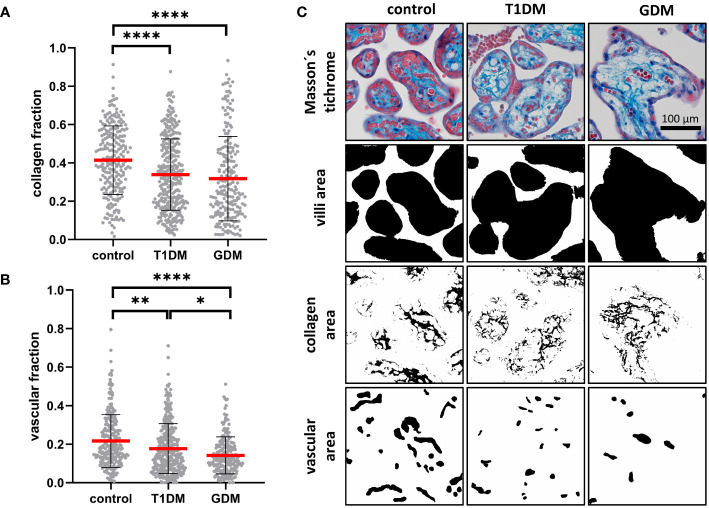
Characterisation of placental villi. **(A, B)** Collagen and vascular fractions of placental villi obtained from normal term placenta (controls), placenta with insulin dependent diabetes mellitus (T1DM) and placenta with gestational diabetes mellitus (GDM). Red line represents mean, error bars represent SD, each point represents one terminal villus. Number of terminal villi: controls (n = 270), T1DM (n = 330), GDM (n = 210). Results were evaluated by Kruskal-Wallis followed by Dunn’s multiple comparison test at a significance level of p< 0.05. Significant results are marked with an asterisk (*) directly in the graphs: * for p< 0.05, ** for p< 0.01, and **** for p< 0.0001. **(C)** Representative micrographs showing Masson’s trichrome staining (collagen is blue) and binary representation of total villus area, collagen area, and vascular area (created with ImageJ software). These areas were measured and used to calculate the collagen and vascular fractions. All micrographs are magnified 200x, black line represents 100 μm.

Masson’s trichrome staining was used to assess the maturity of the placental terminal villi. The collagen fraction was highest in controls and significantly different from both T1DM (p< 0.0001) and GDM (p< 0.0001). There was no difference between T1DM and GDM (p = 0.5852). Means ± SD for controls, T1DM and GDM were plotted: 0.4140 ± 0.1789, 0.3388 ± 0.1868 and 0.3176 ± 0.2202, respectively. The vascular fraction was also highest in controls and significantly different from both T1DM (p = 0.0002) and GDM (p< 0.0001). Moreover, there was significant difference between T1DM and GDM (p = 0.0235), GDM showed the lowest values with a mean of 0.1415 ± 0.0962 (T1DM: 0.1766 ± 0.1300, controls: 0.2162 ± 0.1373). For results see **Figure 4**.

## Discussion

We investigated the phenotype and expression profiles of IL-1β, IL-10, CYP2C8, CYP2C9, CYP2J2 and sEH in HBCs from patients with T1DM and GDM compared to healthy controls. To our knowledge, this is the first study that directly compares these parameters in two types of diabetes and healthy controls. We used immunohistochemistry to detect antigens of interest in HBCs in situ. This approach eliminates contamination by macrophages of maternal origin.

Diabetes is known to be closely associated with inflammation (43). Our work confirmed that HBCs maintain the M2 phenotype in both T1DM and GDM by expressing CD206. This is consistent with previous studies describing that HBCs maintain the M2 phenotype in GDM (44, 45) as well as in uncontrolled T2DM (46). Conversely, other authors have described a decrease in the M2 phenotype with an increase in the M1 phenotype in both GDM and T1DM (28, 43). However, it is not clear whether Pan et al. only determined HBCs or also included decidual marcophages. Sisino et al. assessed the M1 phenotype based on CD68 positivity, although according to current opinion, CD68 antigen is considered as pan-macrophage marker and not specific for the M1 phenotype.

Although HBCs maintained the M2 phenotype, they expressed IL-1β. We found that the two types of diabetes differed significantly in IL-1β expression. In HBCs, IL-1β was significantly upregulated only in T1DM and remained unchanged in GDM. This is consistent with previous findings (28, 41). Sisino et al. demonstrated a significant increase in the expression of proinflammatory cytokines, including IL-1β, in T1DM (28). Schliefsteiner et al. showed that the cytokines IL-1β, TNFα, IL-6 and IL-10 were not significantly altered in GDM compared to controls (41). Although we found a difference in the expression of IL-1β between T1DM and GDM, the level of the anti-inflammatory cytokine IL-10 in HBCs was comparable in all groups tested. This may suggest that immunological tolerance at the feto-maternal interface is not affected. IL-10 plays an essential role in the establishment and maintenance of pregnancy and prevents preterm labour, which is often associated with inflammation. In addition, IL-10 regulates immune cell phenotypes and functions by modulating oxidative phosphorylation and glycolysis (47, 48).

According to some authors, there is a strong possibility that in diabetes, HBCs may undergo an inter-subgroup conversion or potentially adopt a novel, distinct phenotype, rather than simply maintaining M2 polarisation or transitioning to the pro-inflammatory M1 phenotype (49). This hypothesis is also supported by the fact that the production of proinflammatory cytokines is not solely associated with the M1 phenotype. This is supported by the IL-1β and IL-10 expression profiles detected in the current study as well as by our previous results clearly demonstrating the colocalization of CD206 and IL-1β expression in HBCs (50). In addition to M1 macrophages, the M2b subtype is also capable of producing proinflammatory cytokines (41). The basic classification into M1 and M2 macrophages seems to be oversimplified. The placenta is an organ where a new classification of macrophages has been proposed, abandoning the M1/M2 division and taking into account more developmental and functional aspects (1).

Among other functions, EETs also regulate the immune response. They are generally regarded as anti-inflammatory molecules. It is known that the level of EETs in tissues is determined by the ratio of the expression of EET-generating enzymes (CYP epoxygenases) and EET-degrading enzymes (sEH) (51). To the best of our knowledge, direct measurement of EETs and DHETs levels from paraffin-embedded tissue samples is not possible. In normal placenta, we found a high expression of CYP epoxygenases accompanied by a low expression of sEH, suggesting higher concentrations of EETs in a given cell type. In T1DM, there is a decrease in the expression of CYP epoxygenases, while the level of sEH does not change. In contrast, in GDM, CYP epoxygenase expression is maintained or decreases slightly, but sEH expression increases significantly. This relationship suggests different levels of EETs in T1DM and GDM.

There is a lack of information in the current literature on the interplay between CYP epoxygenases and interleukins in HBCs. The only paper suggesting a relationship between IL-1β, IL-10 and CYP epoxygenases is our previous work (50) in which we studied normal embryonic, early fetal and term placentas. We identified CYP2C8 as a key epoxygenase in HBCs. We demonstrated a strong positive correlation of CYP2C8 expression with IL-10 and a strong negative correlation of CYP2C8 expression with IL-1β during the study period. In current study, we found low expression of CYP epoxygenases and barely detectable sEH in HBCs together with inflammatory activation in T1DM. In GDM, the expression levels of CYP epoxygenases were not significantly affected compared to control, suggesting preserved EET synthesis. However, sEH expression was significantly increased, suggesting the occurence of DHET metabolites.

In addition to HBCs, the relationship between cytokines and EETs has been established in other tissue macrophages. Recently, both EETs and sEH inhibition have been shown to reduce the expression of NLRP3 inflammasome-related molecules, suppress calcium overload and reactive oxygen species (ROS) production in macrophages (52). Interestingly, Bystrom et al. conclude that epoxygenases are immunomodulators that regulate macrophages depending on the underlying activation state (33). In the case of classical macrophage activation (M1), inhibition of epoxygenases led to an increase in TNFα and COX-2 expression, which was reversed by the addition of exogenous 11,12-EETs. Surprisingly, in the case of alternative activation (M2), inhibition of epoxygenases led to a decrease in TNFα. In mouse models, EETs attenuated the M1 phenotype and the production of pro-inflammatory cytokines such as IL-1β, IL-6 and TNFα, while preserving the M2 phenotype (29, 34).

However, the role of DHETs in the regulation of inflammation is not clear as they have been studied less than EETs. It has been shown that 5,6-EET and, surprisingly, 5,6-DHET downregulate IL-6 production in macrophages (53). In addition, Diclofenac increased 5,6-DHET levels in obesity-related inflammatory states, suggesting its anti-inflammatory role (54). A recent study by Bergmann et al. investigated the role of 14,15-DHET in the inflammatory response in burn injured mice. They found that 14,15-DHET functionally impairs neutrophil activation, ROS production, acidification and the expression of CXCR1 and CXCR2, the latter potentially inhibiting migration. This impairs the function of neutrophils as key elements of innate immunity. They also showed that DHET administration did not lead to a change in systematic IL-6 levels. This suggests that the increase in EETs and not the decrease in DHETs may be responsible for the decrease in IL-6 (55). Information mentioned above could explain the differences we detected in the inflammatory activation of HBCs in T1DM and GDM. As the exact role of DHETs in inflammation is not fully understood, further research is needed in this area, particularly in the tissue context.

One of the major roles of HBCs during normal pregnancy is their involvement in the process of placentation, specifically promoting angiogenesis, extracellular matrix remodelling, growth, and villous branching. This is suggested by the demonstration of VEGF, MMP-9 and TIMP-1 expression in HBCs (1). Diabetes manifests itself by delayed maturation of placental villi and their pathological vascularisation (31, 32). We confirmed it by finding a lower collagen fraction and hypovascularisation in placentas with T1DM and GDM compared to controls. Although collagen content was comparable in both types of diabetes, interesting results were obtained for vascularisation. We detected more pronounced hypovascularization in GDM than in T1DM. Given the described IHC profiles of CYP epoxygenases and sEH in HBCs in T1DM and GDM, the causes of hypovascularization may be different. EETs are known to be proangiogenic molecules. The low expression of CYP epoxygenases in HBCs in T1DM suggests that their concentrations are low, which could lead to a decrease in vascularisation. On the other hand, the expression of CYP epoxygenases is preserved in GDM. However, the high expression of sEH in HBCs in GDM could lead to EET hydrolysis and the presence of DHET metabolites. The exact role of DHET metabolites in angiogenesis is not well understood. 56 suggested that DHETs are inactive in terms of angiogenesis (56). More recently, Kumar et al. suggested that high levels of DHETs may reduce Notch signalling in angiogenesis (57). However, more research confirming potential anti-angiogenic effect of DHETs is needed since sEH could be pharmacologically targeted.

Generally, it seems that a high level of EETs is desirable in HBCs. EETs promote the M2 phenotype of macrophages, limit inflammation, help overcome insulin resistance and improve glucose tolerance, and influence angiogenesis (58, 59). Our findings may have potential implications for the pharmacological management of different types of diabetes during pregnancy. Currently available analogues of EETs (60) and in particular sEH inhibitors are in clinical trials (61, 62). The use of EET analogues may have a beneficial effect, particularly in T1DM. On the other hand, pharmacological inhibition of sEH seems to be a suitable approach in GMD. Further research in this area is therefore highly desirable.

Taken together, T1DM, we demonstrated inflammatory activation of HBCs based on high IL-1β expression accompanied by a marked decrease in CYP epoxygenase expression compared to normal term placenta. In contrast, inflammatory activation of HBCs is unlikely to occur in GDM, as the expression of both IL-1β and CYP epoxygenases does not change significantly compared to normal placenta. However, in contrast to T1DM and normal placenta, there is a significant increase in sEH expression. This suggests a possible link between the CYP epoxygenase-EETs-sEH axis and IL-1β expression, confirming its anti-inflammatory effect. In both types of diabetes, we observed a delayed maturation of placental villi and their hypovascularization, which was more pronounced in GDM than in T1DM. Based on the different expression profiles of HBCs between T1DM and GDM, a different pharmacological approach to influence placental structure and function seems to be desirable. Given the stable high expression of IL-10 in both controls and both types of diabetes, it appears that immune tolerance is maintained in HBCs.

## Data availability statement

The raw data supporting the conclusions of this article will be made available by the authors, without undue reservation.

## Ethics statement

The studies involving humans were approved by Ethics Committee of the University Hospital and the Faculty of Medicine and Dentristy, Palacký University, Olomouc. The studies were conducted in accordance with the local legislation and institutional requirements. The participants provided their written informed consent to participate in this study.

## Author contributions

ZT: Investigation, Writing – original draft. AB: Data curation, Investigation, Writing – review & editing. KK: Formal analysis, Investigation, Validation, Writing – review & editing. MM: Data curation, Investigation, Writing – review & editing. MJ: Formal analysis, Writing – review & editing. KC: Conceptualization, Data curation, Methodology, Supervision, Visualization, Writing – review & editing.
